# 3D Spheroids Facilitate Differentiation of Human Adipose-Derived Mesenchymal Stem Cells into Hepatocyte-Like Cells via p300-Mediated H3K56 Acetylation

**DOI:** 10.1093/stcltm/szad076

**Published:** 2023-11-04

**Authors:** Yanrong Yu, Haina Huang, Junsong Ye, Yumei Li, Renjian Xie, Liping Zeng, Yushan Huang, Tai Zeng, Dan Luo, Jianing Zhong, Weijie Peng

**Affiliations:** Key Laboratory of Biomaterials and Biofabrication for Tissue Engineering in Jiangxi Province, Gannan Medical University, Ganzhou, People’s Republic of China; Center for Molecular Diagnosis and Precision Medicine, and Department of Clinical Laboratory, The First Affiliated Hospital of Nanchang University, Nanchang, People’s Republic of China; Key Laboratory of Biomaterials and Biofabrication for Tissue Engineering in Jiangxi Province, Gannan Medical University, Ganzhou, People’s Republic of China; Key Laboratory of Prevention and Treatment of Cardiovascular and Cerebrovascular Diseases, Ministry of Education, Gannan Medical University, Ganzhou, People’s Republic of China; Key Laboratory of Biomaterials and Biofabrication for Tissue Engineering in Jiangxi Province, Gannan Medical University, Ganzhou, People’s Republic of China; Key Laboratory of Prevention and Treatment of Cardiovascular and Cerebrovascular Diseases, Ministry of Education, Gannan Medical University, Ganzhou, People’s Republic of China; Subcenter for Stem Cell Clinical Translation, The First Affiliated Hospital of Gannan Medical University, Ganzhou, People’s Republic of China; Key Laboratory of Biomaterials and Biofabrication for Tissue Engineering in Jiangxi Province, Gannan Medical University, Ganzhou, People’s Republic of China; School of Basic Medicine, Gannan Medical University, Ganzhou, People’s Republic of China; Key Laboratory of Biomaterials and Biofabrication for Tissue Engineering in Jiangxi Province, Gannan Medical University, Ganzhou, People’s Republic of China; School of Medical Information Engineering, Gannan Medical University, Ganzhou, People’s Republic of China; Key Laboratory of Biomaterials and Biofabrication for Tissue Engineering in Jiangxi Province, Gannan Medical University, Ganzhou, People’s Republic of China; Center for Evidence Based Medical and Clinical Research, The First Affiliated Hospital of Gannan Medical University, Ganzhou, People’s Republic of China; Key Laboratory of Biomaterials and Biofabrication for Tissue Engineering in Jiangxi Province, Gannan Medical University, Ganzhou, People’s Republic of China; Department of Physiology, School of Basic Medicine, Nanchang University, Nanchang, People’s Republic of China; Key Laboratory of Biomaterials and Biofabrication for Tissue Engineering in Jiangxi Province, Gannan Medical University, Ganzhou, People’s Republic of China; Key Laboratory of Prevention and Treatment of Cardiovascular and Cerebrovascular Diseases, Ministry of Education, Gannan Medical University, Ganzhou, People’s Republic of China; Key Laboratory of Biomaterials and Biofabrication for Tissue Engineering in Jiangxi Province, Gannan Medical University, Ganzhou, People’s Republic of China; Key Laboratory of Prevention and Treatment of Cardiovascular and Cerebrovascular Diseases, Ministry of Education, Gannan Medical University, Ganzhou, People’s Republic of China

**Keywords:** human adipose-derived mesenchymal stem cells, 3D spheroids, hepatogenic differentiation, H3K56 acetylation, p300

## Abstract

Hepatocyte-like cells (HLCs) that are differentiated from mesenchymal stem cells (MSCs) provide a valuable resource for drug screening and cell-based regeneration therapy. Differentiating HLCs into 3D spheroids enhances their phenotypes and functions. However, the molecular mechanisms underlying MSCs hepatogenic differentiation are not fully understood. In this study, we generated HLCs from human adipose-derived mesenchymal stem cells (hADMSCs) in both 2D and 3D cultures. We performed an acetyl-proteomics assay on the HLCs derived from both 2D and 3D differentiation and identified a differential change in H3K56 acetylation between the 2 differentiated cells. Our findings revealed that 3D differentiation activated ALB gene transcription by increasing the acetylation level of H3K56, thereby enhancing the phenotypes and functions of HLCs and further promoting their maturation. Notably, inhibiting p300 reduced the acetylation level of H3K56 during hepatogenic differentiation, leading to decreased phenotypes and functions of HLCs, whereas activation of p300 promoted hepatogenic differentiation, suggesting that p300 plays a critical role in this process. In summary, our study demonstrates a potential mechanism through which 3D spheroids differentiation facilitates hADMSCs differentiation into HLCs by promoting p300-mediated H3K56 acetylation, which could have significant clinical applications in liver regeneration and disease modeling.

## Graphical Abstract

**Graphical Abstract:**
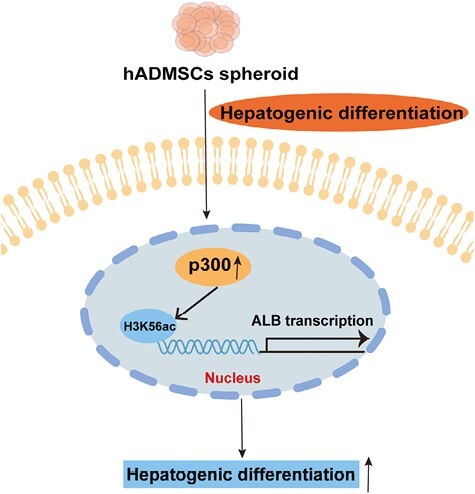

**Significance Statement**

This study has demonstrated that 3D spheroids culture effectively promotes the differentiation of hADMSCs into HLCs. This is achieved by upregulating the acetylation level of H3K56, which is mediated by p300. This, in turn, leads to the activation of ALB transcription and an enhancement of the phenotypes and functions of HLCs. Overall, this novel epigenetic regulatory mechanism offers valuable insights into the process of MSCs hepatogenic differentiation and provides a strong theoretical foundation for the clinical applications of MSCs 3D hepatogenic differentiation.

## Introduction

Liver diseases, such as viral hepatitis, cirrhosis, and hepatocellular carcinoma, have become a leading cause of death worldwide.^1-3^ Orthotopic liver transplantation (OLT) remains the only effective treatment for end-stage liver disease.^4,5^ However, the shortage of available organs, high costs, risks of transplant rejection, and the need for lifelong immunosuppression are major issues, necessitating the search for alternative regenerative approaches.^6,7^ In recent years, mesenchymal stem cells (MSCs) differentiated into hepatocytes have been considered as an effective alternative treatment for liver disease.^8-10^ MSCs have several advantages, including wide availability, multipotent differentiation, low immunogenicity, and immunomodulatory properties,^11,12^ which can be obtained from various sources such as bone marrow, umbilical cord, placenta, and adipose tissue.^13-15^ Human adipose tissue-derived mesenchymal stem cells (hADMSCs) are non-tumorigenic, ethically acceptable, minimally invasive, and easy to isolate.^16^ They are widely used for differentiation into hepatocyte-like cells (HLCs), as ideal seed cells for hepatocyte therapy and liver regeneration medicine.^17^ Hepatogenic differentiation of hADMSCs has mainly been induced under 2-dimensional (2D) culture.^18,19^ However, 2D culture is not optimal for hepatocytes because it cannot mimic the 3-dimensional (3D) structure of the liver. With the advancement of 3D culture techniques, an increasing number of studies are focusing on 3D hepatogenic differentiation. 3D spheroids, as a 3D culture approach, promote cell-cell and cell-ECM interactions, creating an “in vivo-like” microenvironment.^20,21^ Many studies suggest that 3D culture promotes hepatogenic differentiation of MSCs.^22-24^ However, the mechanisms underlying the promotion of hepatogenic differentiation of MSCs through 3D culture remain to be further elucidated.

Histone acetylation is a post-translational modification of histones, mediated by histone acetyltransferases (HATs) and histone deacetylases (HDACs), that mainly influences acetylation of lysine residues, which are involved in the activation of gene transcription.^25,26^ Histone acetylation modifications are closely associated with both 2D and 3D cell cultures.^27^ A study has shown that histone-modifying enzymes change in MSCs under 3D culture conditions.^28^ Histone acetylation has been shown to play a crucial role in the pluripotency and differentiation of MSCs.^29,30^ Studies have indicated that 3D spheroids culture of MSCs increases their multipotency and alters the histone acetylation status on their pluripotent genes.^31,32^ Studies have shown that inhibiting HDACs promotes the hepatogenic differentiation of human bone marrow mesenchymal stem cells.^33,34^ Inhibiting HDACs can promote the hepatogenic differentiation of hADMSCs and improve their hepatic functional characteristics.^35^ Therefore, it is hypothesized that histone acetylation may be involved in the regulation of hepatogenic differentiation of hADMSCs under 3D culture conditions, and further studies are needed to elucidate the underlying mechanisms.

Histone H3 lysine 56 acetylation (H3K56ac) is a modification located in the globular core of the histone octamer that was first identified in yeast in recent years.^36^ Acetylation of H3K56 can loosen the wrapping of DNA, thereby altering the higher-order structure of chromatin and recruitment of effector proteins, ultimately leading to transcription of downstream genes and participation in DNA replication and DNA damage repair.^37^ Studies have shown that H3K56 acetylation regulates the expression of pluripotency transcription factors in human embryonic stem cells (hESCs).^38^ H3K56 acetylation is also found to interact with Oct4, promoting the transcriptional activation of pluripotency genes and the differentiation of ESCs.^39^ Although many studies have been conducted on H3K56 acetylation of stem cells stemness, few studies specifically evaluate the role of H3K56 acetylation in hepatogenic differentiation of MSCs. p300 is a transcriptional coactivator with HATs enzymic activity that regulates the acetylation of histone H3 at lysine 9, lysine 14, and lysine 56 and other sites, and plays a critical role in transcriptional regulation and cellular processes.^40-42^ However, there is limited knowledge about the roles of p300 in regulating H3K56 acetylation in hepatogenic differentiation of hADMSCs under 2D and 3D culture conditions.

In this study, we performed an acetylation proteomics assay on HLCs derived from both 2D and 3D differentiation and determined the differential change in H3K56 acetylation in these 2 differentiated cells. Our work found that p300 modulates hADMSCs hepatogenic differentiation in 2D and 3D cultures through regulation of H3K56 acetylation. 3D differentiation, by upregulating the level of H3K56 acetylation and activating ALB transcription, enhances the phenotypes and functions of HLCs, further promoting the maturation of HLCs.

## Materials and Methods

### Isolation and Culture of hADMSCs

hADMSCs were isolated from human female voluntary donated adipose tissue obtained from The First Affiliated Hospital of Gannan Medical University. Briefly, the adipose tissue was first washed several times with PBS buffer containing 1% penicillin/streptomycin (Solarbio, China), then the adipose tissue was minced into small pieces and digested with 1g/L Collagenase type I (Sigma-Aldrich, USA) at 37 ℃ for 60 minutes with gentle shaking at 100 rpm. After tissue digestion was completed, added MEM-α (BI, Israel) complete culture medium containing 10% FBS (Gibco, USA) and 1% penicillin/streptomycin to terminate digestion and collect the cell suspension through a 100-μm cell strainer (BD Falcon, USA). The cell suspension was subsequently centrifuged at 1200 rpm for 5 minutes, and then the cell pellet was resuspended in MEM-α medium containing 15% FBS and 1% penicillin/streptomycin. The cells were cultured in a cell culture dish at 37 °C with a humidified atmosphere of 5% CO_2_. The medium was changed every 3 days. After reaching 80%-90% confluence, the cells were passaged with 0.25% TrypLE Express Enzyme solution (Gibco) and used at passage 3 in all subsequent experiments. This study was approved by the Ethics Committee of the First Affiliated Hospital of Gannan Medical University. The obtained tissue samples were only used for scientific research. Informed consent was obtained from the donators who voluntarily donated their adipose tissue prior to participation.

### Flow Cytometry Analysis

hADMSCs (1 × 10^6^ cells/mL) were washed and resuspended in staining buffer (PBS). Cells were stained with PE-conjugated antibodies against human CD44, CD90, and HLA-DR; FITC-conjugated antibodies against human CD34, CD45, CD105, and their isotype controls (antibodies all from BD Biosciences), incubated in the dark at 2-8 °C for 30 minutes. The cell suspensions were washed twice and resuspended in 500 μL PBS for flow cytometry (FACS Canto II, BD Biosciences) using FLOWJO version 10 software (Becton, Dickinson and Company).

### Osteogenic and Adipogenic Differentiation

Passage 3 hADMSCs were seeded at a density of 2 × 10^4^ cells/cm^2^ in a 6-well plate pre-coated with 0.1% gelatin. When the cells reached 70%-80% confluence, MSC osteogenic differentiation medium (Cyagen Biosciences) was added to the wells according to the manufacture’s instruction, while complete culture medium (MEM-a) was added to the other wells as a negative control. The medium was changed every 3 days for 3 weeks. After 21 days, Alizarin Red (pH 4.2, 40 mM) staining was performed to assess the osteogenic differentiation potential. For adipogenic differentiation, hADMSCs reached 100% confluence, MSC adipogenic differentiation medium (Cyagen Biosciences) was added to the wells according to the manufacturer’s instruction, while complete culture medium was added to the other wells as a negative control. After induction, intracellular lipid droplet formation was assessed using Oil Red O staining (Solarbio) to evaluate the adipogenic differentiation potential.

### 3D Spheroids Culture of hADMSCs

Spheroids were generated on agarose-based microwells fabricated using 3D printing technology and agarose molded technology. Briefly, 2% warm agarose solution (Sigma-Aldrich) was added to each well of a 12-well plate, and a mold was placed into agarose solution. Then, when the agarose solidified, gently removed the mold to form an array of 450 microwells with a diameter of 200 μm each. All agarose microwells were washed with PBS. hADMSCs were seeded onto the agarose microwells at a density of 1 × 10^6^ cells/mL, with 1 mL added to each well of 12-well plate. Then the 12-well plate was centrifuged at 300*g* for 3 minutes and placed in an incubator supplied with a humidified atmosphere of 5% CO_2_ at 37 °C. The next day, uniform 3D cell spheroids were formed in each microwell.

### Hepatogenic Differentiation of hADMSCs in 2D and 3D Culture Conditions

Hepatogenic differentiation was performed by using a 2-step protocol.^43^ For 2D culture differentiation, 3th-5th passage hADMSCs were seeded on 0.1% gelatin-coated dishes at a density of 3 × 10^4^ cells/cm^2^ and cultured in growth medium at 37 °C with 5% CO_2_. When the cells reached 100% confluence, the cells were serum deprived for 2 days with Iscove’s Modified Dulbecco’s Medium (IMDM; Gibco) supplemented with 20 ng/mL EGF (Peprotech, USA) and 10 ng/mL bFGF (Peprotech). Hepatoblast cells differentiation was induced by treating hADMSCs with IMDM supplemented with 20 ng/mL HGF (Peprotech) and 10 ng/mL bFGF, 0.61 g/L nicotinamide (Sigma-Aldrich) for 7 days. Hepatocyte-like cells maturation differentiation was treated with IMDM supplemented with 20 ng/mL oncostatin M (Peprotech), 1 μM dexamethasone (Sigma-Aldrich), and 1 × ITS^+^ (Sigma-Aldrich) for 2 weeks, medium changes were performed twice weekly. For 3D culture differentiation, as described above, after hADMSCs were formed 3D cell spheroids, 3D hepatogenic differentiation was performed also according to the 2-step protocol. For p300 regulated H3K56 acetylation affecting hepatogenic differentiation of hADMSCs experiments, p300 inhibitor A-485 (3 μM, MedChemExpress, USA) and activator CTB (10 μM, MedChemExpress), according to previous reports,^44-47^ were added in hepatocyte-like cells maturation stage (days 7-21) during 2-step process. A-485-1 group was 3 μM A-485 was added on days 7-14 during HLCs maturation stage, A-485-2 group was 3 μM, A-485 was added on days 14-21 during HLCs maturation stage, A-485-3 group was 3 μM, A-485 was added on days 7-21 during HLCs maturation stage.

### Live/Dead Staining

A fluorescent live/dead assay kit (Yeasen BioTech, China) was used to determine the cell viability after hepatogenic differentiation of hADMSCs in 2D and 3D culture conditions following the manufacturer’s instruction. Briefly, HLCs differentiated from hADMSCs and undifferentiation hADMSCs under different culture dimensions (2D, 3D) were incubated in 5 mL of 1 × assay buffer mixed solution containing 15 µL of propidium iodide (1.5 mM) and 5 µL of Calcein-AM (2 mM) at 37 °C in the dark for 30 minutes. The images were acquired by a fluorescence microscope (LEICA, Germany). Green staining represents live cells and red staining represents dead cells.

### RNA Extraction and Real-Time Quantitative PCR

For 3D cells, sonication at 25% output (Power) on ice for 30 s was required before adding Trizol reagent to extract RNA. Total RNA was extracted using Trizol reagent (Invitrogen, USA). Total RNA (1 μg) was reverse transcribed into cDNA with a Reverse Transcription Kit (TransGen Biotech, China) according to the manufacturer’s protocol. Real-time quantitative PCR was performed by mixing cDNA, gene-specific primers, and 2× Top/Tip Green qPCR SuperMix (TransGen Biotech) in the QuantStudio 1 Real-Time PCR System (Applied Biosystems). Three biological replicates were measured. GAPDH was used as an internal standard, and the relative abundance fold changes of each target gene was determined using the 2^−ΔΔCt^ formula. Primer sequences are provided in Table 1.

**Table 1.** Forward and reverse primer pairs used for polymerase chain reactions to detect hepatic specific gene and stemness gene transcripts.

Gene	Forward primer 5ʹ-3ʹ	Reverse primer 5ʹ-3ʹ
*GAPDH*	GGAGCGAGATCCCTCCAAAAT	GGCTGTTGTCATACTTCTCATGG
*Sox2*	TGGACAGTTACGCGCACAT	CGAGTAGGACATGCTGTAGGT
*Oct4*	CTTGAATCCCGAATGGAAAGGG	CCTTCCCAAATAGAACCCCCA
*Nanog*	AAGGTCCCGGTCAAGAAACAG	CTTCTGCGTCACACCATTGC
*ALB*	TGCAACTCTTCGTGAAACCTATG	ACATCAACCTCTGGTCTCACC
*AFP*	AGTGAGGACAAACTATTGGCCT	ACACCAGGGTTTACTGGAGTC
*CK18*	TCGCAAATACTGTGGACAATGC	GCAGTCGTGTGATATTGGTGT
*CK19*	ACCAAGTTTGAGACGGAACAG	CCCTCAGCGTACTGATTTCCT
*HNF-4α*	CACGGGCAAACACTACGGT	TTGACCTTCGAGTGCTGATCC
*CYP1A2*	ATGCTCAGCCTCGTGAAGAAC	GTTAGGCAGGTAGCGAAGGAT
*CYP3A4*	AAGTCGCCTCGAAGATACACA	AAGGAGAGAACACTGCTCGTG

### Western Blot Analysis

Cells were lysed in RIPA buffer (Solarbio) supplemented with complete protease inhibitors (Thermo Fisher, USA) on ice for 10 minutes. For 3D cells, sonication at 25% output on ice for 30 s was required before centrifugation, cell lysates were collected and centrifuged at 12 000 rpm for 15 minutes at 4 °C. Protein concentration in the supernatant was determined by the BCA protein assay kit (Beyotime, China). Following the addition of protein loading buffer (Solarbio), the samples were boiled at 95 °C for 10 minutes. 30 μg proteins were separated by 12% SDS-PAGE gel and transferred to 0.45 μm polyvinylidine difluoride (PVDF) membranes (Millipore, USA). Following blocking with 5% non-fat milk for 2 hours at room temperature, the membranes were incubated with primary antibodies at 4 °C overnight, which were anti-Sox2 rabbit polyclonal (1:1000, Abcam, USA), anti-Oct4 rabbit polyclonal (1:1000, Abcam), anti-Nanog rabbit polyclonal (1:1000, Abcam), anti-Histone H3 acetyl K56 rabbit monoclonal (1:1000, Abcam), anti-ALB mouse monoclonal (1:1000, Proteintech, USA), anti-AFP rabbit polyclonal (1:1000, Proteintech), anti-GAPDH mouse monoclonal (1:2000, Proteintech), and anti-H3 rabbit polyclonal (1:1000, Proteintech). The next day, the membranes were washed 3 times with Tris buffered saline with 0.05% Tween 20 (TBST) and then incubated with HRP-conjugated anti-rabbit or anti-mouse secondary antibodies (Cell Signaling Technology, USA) for 1 hour at room temperature. After washing by TBST 3 times, the membranes were visualized using an enhanced chemiluminescence system (Thermo Fisher).

### Immunofluorescence Staining

The cells were fixed with 4% paraformaldehyde (PFA; Solarbio) for 20 minutes at room temperature, and then permeabilized with 0.2% Triton X-100 (Solarbio) for 5 minutes, blocked with 1% bovine serum albumin (BSA; Solarbio) in PBS for 1 hour, and then washed 3 times with PBS. Cells were incubated with mouse anti-ALB antibody (1:200, Proteintech), rabbit anti-AFP antibody (1:200, Proteintech), and rabbit anti-H3K56ac antibody (1:200, Abcam) overnight at 4 °C. The next day, samples were washed 3 times with PBS, and then incubated with FITC conjugated goat anti-mouse IgG (H + L), secondary antibody (1:500, Boster, China), and Dy550 conjugated goat anti-rabbit IgG (H + L) secondary antibody (1:500, Boster, Wuhan, China) for 2 hours at room temperature in the dark. The cells were washed 3 times with PBS, and the nuclei were stained with DAPI solution (Boster) and then imaged using a laser confocal microscope (Carl Zeiss, Germany).

### Albumin Secretion by ELISA

The concentration of albumin in supernatants of hepatocyte-like cells differentiated from hADMSCs after 21 days was determined using Enzyme-Linked Immunosorbent Assay Kit for Albumin (Cloud-Clone Corp, China) according to the manufacturer’s instructions. The corresponding concentration was calculated based on the normalization of the number of cells.

### Urea Detection

To determine the secretion of urea, supernatants of the hepatocyte-like cells differentiated from hADMSCs after 21 days were collected. The concentration of urea of supernatants was measured by QuantiChrom Urea Assay Kit according to the manufacturer’s instructions. The corresponding concentration was calculated based on the normalization of the number of cells.

### Periodic Acid-Schiff (PAS) Staining

The cells were washed with PBS 3 times, and then fixed with 4% PFA for 15 minutes. After fixation, cells were incubated with 1% periodic acid (Sigma, USA) for 10 minutes, and then washed with distilled water prior to incubation with Schiff’s reagent (Sigma) at 37 °C for 20 minutes. After a 5-minute wash in tap water, cells were washed and visualized under a light microscope (Olympus, Japan), PAS staining was performed to detect glycogen in liver cells. Glycogen appeared as a purplish-red stain, while the nuclei were stained blue. Positive PAS staining exhibited a more intense purplish-red color.

### Acetylation Proteomics Analysis

To determine the differentially expressed histones of acetylation modification of HLCs in 2D and 3D differentiation, 2D-HLCs and 3D-HLCs were subjected to acetylation modified proteomics detection (PTM Biolabs, Hangzhou, China). Proteins of 2D-HLCs and 3D-HLCs were extracted in lysis buffer (8 M urea, 1% protease inhibitor cocktail, 3 μM TSA, 50 mM NAM) and quantified using BCA kit according to the manufacturer’s instructions, and then the proteins were added to trypsin at a 1:50 trypsin-to-protein mass ration for digestion. Tryptic peptides were incubated with pre-washed antibody beads and enriched with pan-PTM (post-translational modification) antibody based, the bound peptides were eluted and vacuum dried. The resulting peptides were desalted with C18 ZipTips (Millipore) according to the manufacturer’s instructions for further LC-MS/MS analysis. The resulting LC-MS/MS data were processed using MaxQuant search engine (v.1.6.15.0). Tandem mass spectra were searched against the human SwissProt database (20 422 entries) concatenated with reverse decoy database. These data were used in subsequent bioinformatics analysis. The screening of differential modification sites followed the following criteria: 1.5 times the change threshold.

### Chromatin Immunoprecipitation-Real Time Quantitative PCR (ChIP-qPCR)

ChIP assay was performed according to the protocol of EpiQuik Chromatin Immunoprecipitation kit (EpigenTek, USA). Briefly, cells (1 × 10^7^) were collected, followed nuclear extracted by cell lysis, and the lysates were sonicated using Sonifier (Active Motif, USA) to obtain chromatin fragments of 200-1000 bp. Chromatins were incubated and precipitated with antibody against H3K56ac or IgG. Cross-linked DNA was reversed and DNA was purified with spin column. Primer sequences to detect the H3K56ac binding site along the ALB promoter were listed as follows: ALB forward, 5ʹ-AGTCTCTGTGCCTCTATGTGCC-3ʹ and reverse, 5ʹ- ACCTCTGCCCTTTTGCTCAGA -3ʹ. In the ChIP-qPCR analyses, the values from the immunoprecipitated samples were normalized to that from the input DNA. PCR amplification products were examined on 1% agarose gel.

### Statistical Analysis

Data are presented as mean ± standard deviation (SD) and were analyzed using GraphPad Prism 7.0 software (GraphPad Software Inc., San Diego, CA, USA). The differences between 2 groups were determined by Student’s *t* test (2-tailed). One-way ANOVA was applied when more than 2 groups were compared. Differences with *P* < 0.05 were considered statistically significant.

## Results

### Characterization of hADMSCs

**Figure 1.**
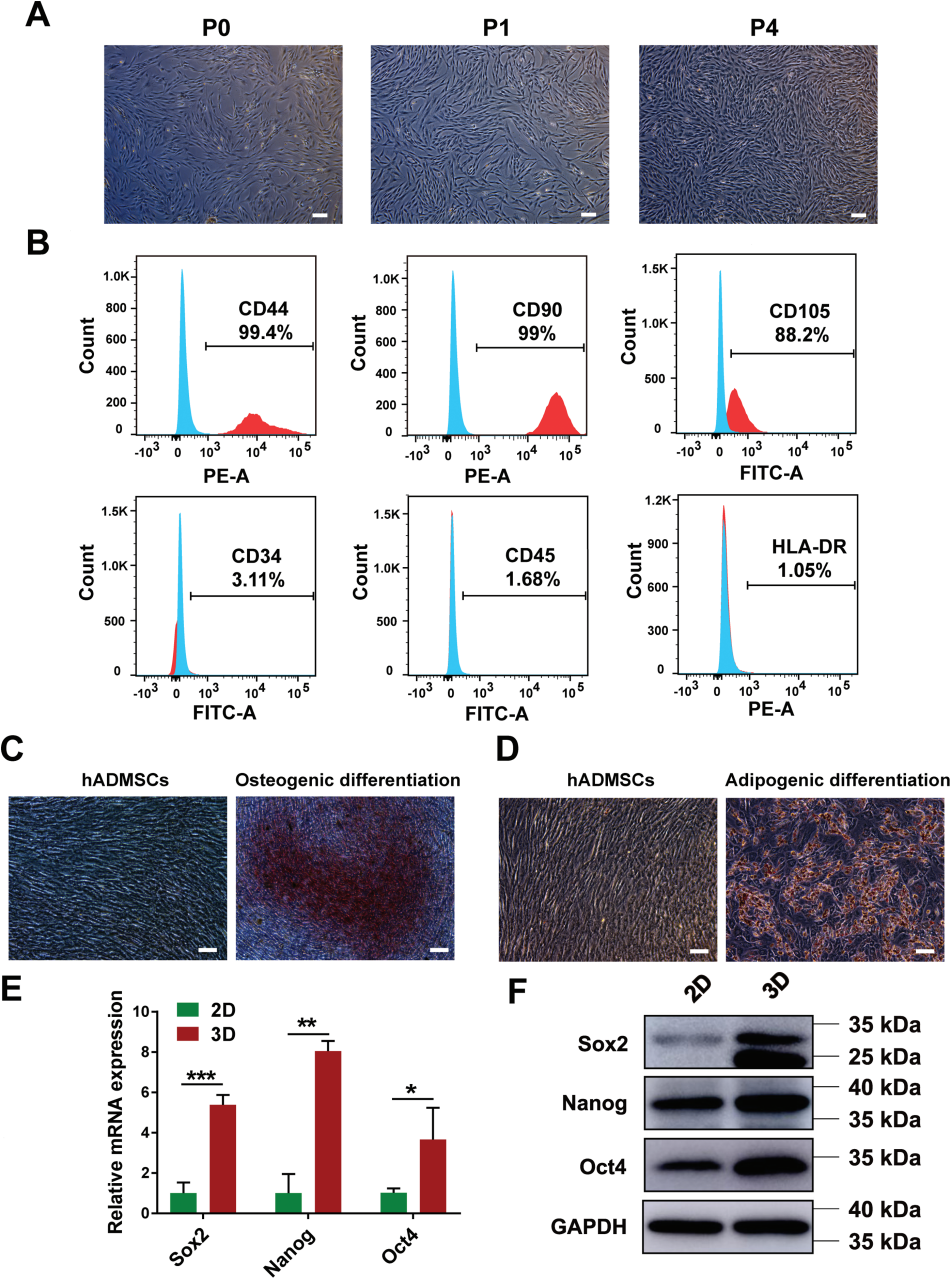
Characterization of human adipose-derived mesenchymal stem cells (hADMSCs). **A**, The morphology of hADMSCs of different passages (passage 0, passage 1, and passage 4). Scale bar = 100 μm. **B**, Expression of cell surface markers on hADMSCs. Cells were incubated with FITC or PE-labelled anti-human-specific antibodies and analyzed by flow cytometry, and cells were positive for CD44, CD90, and CD105 and negative for CD34, CD45, and HLA-DR. **C**, Osteogenic differentiation potential of hADMSCs was demonstrated by staining with Alizarin Red. Scale bar = 100 μm. **D**, Adipogenic differentiation potential of hADMSCs was confirmed by Oil Red O staining. Scale bar = 100 μm. **E**, The mRNA expression of pluripotent transcription factors (Sox2, Nanog and Oct4) of hADMSCs in 2D and 3D cultures by RT-qPCR. *P < .05, **P < .01, ***P < .001. **F**, The protein expression of pluripotent transcription factors of hADMSCs in 2D and 3D cultures by western blot. Data are presented as mean ± SD.

hADMSCs exhibited spindle-shaped fibroblast-like morphology and rapid proliferation, reaching 80%-90% confluence in approximately 3-4 days (Fig. 1A). Flow cytometry analysis revealed that hADMSCs were positive for the mesenchymal markers CD44, CD90, and CD105, and negative for hematopoietic markers CD34 and CD45. Low immunogenicity was also observed, as hADMSCs exhibited low expression of HLA-DR (Fig. 1B). Osteogenic and adipogenic differentiation potential was confirmed by Oil Red O and Alizarin Red staining, respectively (Fig. 1C, 1D). These results indicate that hADMSCs extracted in this study exhibited typical characteristics of MSCs and had pluripotent differentiation potential. Pluripotency transcription factors Sox-2, Oct4, and Nanog were analyzed by RT-qPCR and Western blot, with results showing upregulation of mRNA and protein expression in 3D spheroids culture compared with 2D-culture (Fig. 1E, 1F). These findings suggest that 3D spheroids formation may facilitate the maintenance of MSCs’ stemness properties.

### hADMSCs Differentiated Into Hepatocyte-Like Cells under 2D and 3D Cultures In Vitro

**Figure 2.**
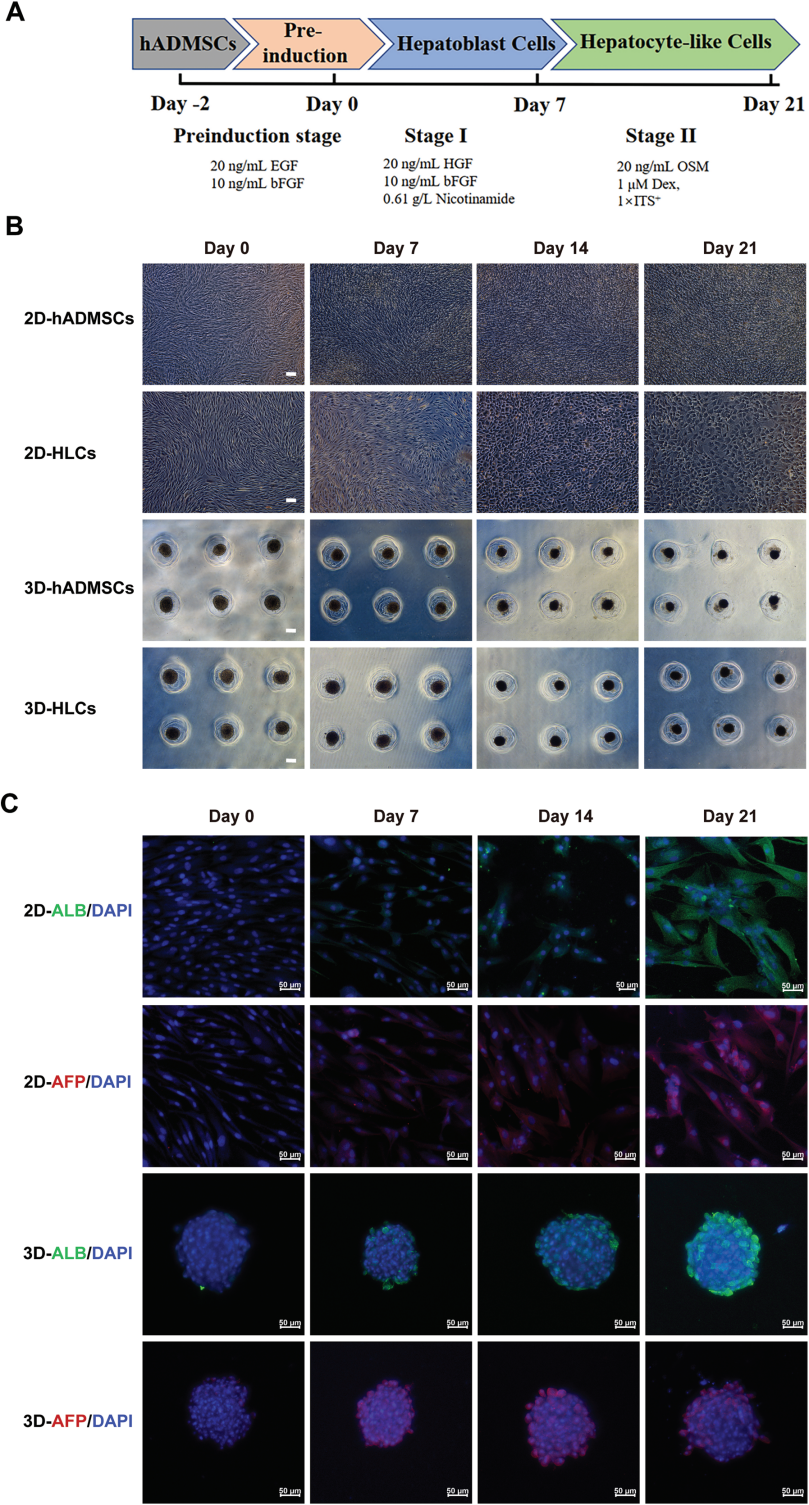
hADMSCs differentiated into HLCs in 2D and 3D cultures in vitro. **A**, Schematic diagram of hADMSC differentiation into HLCs. **B**, The sequential morphological changes of 2D and 3D hADMSCs induction and non-induction at different time points during hepatic induction. Scale bar = 100 μm. **C**, The protein expression of ALB, AFP of hADMSCs differentiated into HLCs at different time points during 2D and 3D hepatic differentiation. Scale bar = 50 μm.

To differentiate hADMSCs into HLCs, a 2-step induction protocol was used for both 2D and 3D cultures (Fig. 2A), following previous reports.^23,43,48^ In the 2D induction group, hADMSCs were seeded on 0.1% gelatin-coated dishes, and after reaching approximately 100% confluence, the cells were treated with hepatic induction medium. During the differentiation process, hADMSCs gradually transformed from spindle and fibroblast-like cells into round or polygonal cells. On day 7, a large number of round-shaped cells were observed in the induction group, which showed growth arrest. By day 14, the majority of cells transformed into polygonal shape, and by the 21st day, the differentiated cells showed further maturation, exhibiting a change in morphology from spindle-shaped fibroblast-like cells to polygonal cells with abundant cytoplasmic granules, compared to normal hADMSCs cultured for 21 days. As for the 3D induction group, the cell spheroids both in the induced and non-induced groups became condensed, with a decrease in diameter from approximately 160 μm before induction to approximately 120 μm after 21 days of induction. However, no significant changes in cell morphology were observed between the induced and non-induced groups due to their spherical morphology (Fig. 2B). The protein expression of liver-related genes during the 21-day induction process was shown in Fig. 2C, with the protein expression of ALB increasing in both 2D and 3D induced cells with induction time. The protein expression of AFP in 2D induced cells increased over time, while the AFP protein expression in 3D induced cells decreased. These results suggest that HLCs derived from hADMSCs in both 2D and 3D cultures exhibited protein expression of ALB and AFP.

### 3D Hepatogenic Differentiation of hADMSCs Enhanced the Phenotypes and Functions of Hepatocyte-Like Cells

**Figure 3.**
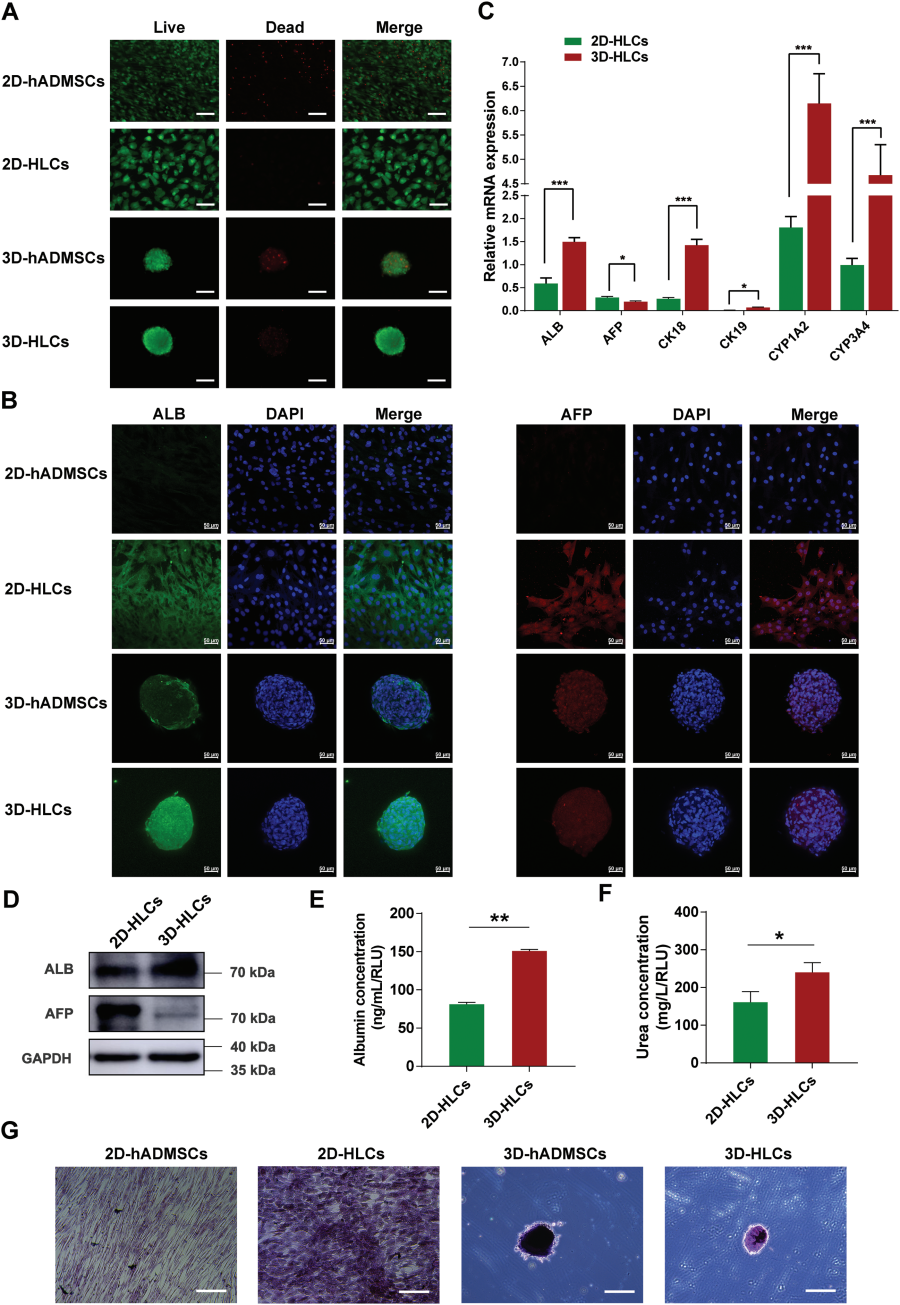
The phenotypes and functions of hepatocyte-like cells (HLCs) differentiated from hADMSCs after 2D and 3D differentiation in vitro. **A**, The fluorogram of live/dead cells staining of hADMSCs and HLCs after 2D and 3D differentiation. hADMSCs as the undifferentiated cells were cultured in hADMSCs normal medium. Scale bar = 100 μm. **B**, The immunofluorescent images of expression of ALB and AFP of hADMSCs and HLCs after 2D and 3D differentiation. Scale bar = 50 μm. **C**, The mRNA expression levels of liver-related gene in 2D-HLCs and 3D-HLCs were determined by RT-qPCR. Data are normalized to GAPDH. *P < .05, ***P < .001. **D**, The protein expression levels of ALB and AFP in 2D and 3D-HLCs were analyzed by western blot. **E**, The albumin secretion in 2D and 3D-HLCs was detected by ELISA. **P < .01. **F**, The urea production in 2D and 3D-HLCs was measured by quantitative colorimetric analysis. *P < .05. **G**, Glycogen was detected by periodic acid-schiff (PAS) staining of undifferentiation cells (hADMSCs) and HLCs after 2D and 3D differentiation. Scale bar = 100 μm. Data are presented as mean ± SD.

We used live/dead staining to detect the cell viability of HLCs derived from hADMSCs after 2D and 3D induction (2D-HLC and 3D-HLCs). As shown in Fig. 3A, both 2D- and 3D-induced HLCs exhibited significantly higher cell viability compared to the non-induced group. To investigate the effects of 3D spheroids culture on the hepatogenic differentiation capabilities of hADMSCs, we evaluated the phenotypes and functions of HLCs differentiated from hADMSCs by mRNA and protein expression analysis of liver-related genes, as well as detection of albumin, urea secretion, and glycogen synthesis. After 21 days of induction, the mRNA expression levels of ALB, CK18, CK19, CYP1A2, and CYP3A4 were significantly higher in 3D-HLCs compared to 2D-HLCs, as shown in Fig. 3C. The protein expression of ALB was significantly higher than that of the non-induced group in both 2D- and 3D-induced HLCs, and the protein expression of ALB in 3D-HLCs was higher than that in 2D-HLCs (Fig. 3B, 3D). The protein expression of AFP was significantly higher in 2D-HLCs compared to the non-induced group, while AFP protein expression was lower in 3D-HLCs compared to the non-induced group (Fig. 3B). The mRNA and protein expression of AFP of 3D-HLCs were all significantly reduced (Fig. 3C, 3D). Functions of hepatocytes, such as albumin secretion, urea generation, and glycogen synthesis, are also important indicators for confirming the successful differentiation of hADMSCs into HLCs. As shown in Fig. 3E, 3F, compared with the 2D-HLCs group, the levels of albumin and urea were significantly higher in the 3D-HLCs group, indicating that HLCs possessed the capacity to produce albumin and urea, and that 3D induction enhanced HLCs functions. In addition to albumin secretion and urea production, HLCs also displayed the function of glycogen synthesis. The PAS staining showed that HLCs exhibited glycogen synthesis compared to non-induced hADMSCs, and that 3D-HLCs had higher glycogen synthesis than 2D-HLCs (Fig. 3G). Together, these results demonstrated that hADMSCs were successfully differentiated into HLCs, and 3D induction enhanced the phenotypes and functions of HLCs, further promoting the maturation of HLCs.

### Proteomics of Acetylation Modifications Analysis Identified H3K56ac as a Key Feature of HLCs From 2D and 3D Hepatogenic Differentiation

**Figure 4.**
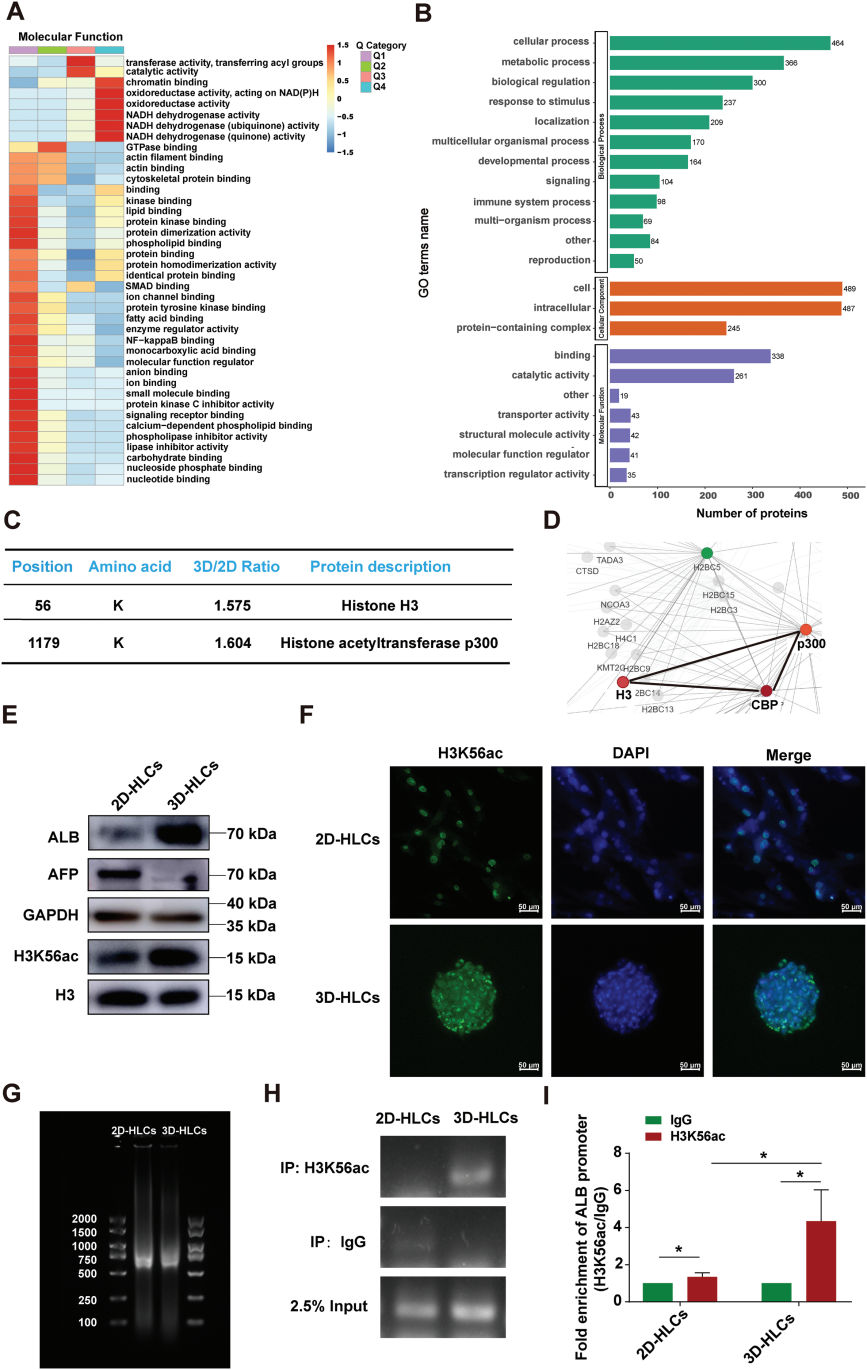
Elevated H3K56 acetylation level promoted ALB gene expression in 3D differentiated-HLCs. **A**, Molecular functional enrichment cluster analysis of proteins with differential acetylation modifications in 2D-HLCs (HLCs differentiated from 2D hADMSCs) and 3D-HLCs (HLCs differentiated from 3D hADMSCs). Four distinct subgroups (Q1, Q2, Q3, and Q4) were defined by cluster analysis of the differentially expressed acetylation proteins based on the fold change in expression between 3D-HLCs and 2D-HLCs. Proteins with a 3D-HLCs/2D-HLCs ratio < 0.5 were categorized as Q1, those with a ratio between 0.5 and 0.667 were classified as Q2, those with a ratio between 1.5 and 2.0 were categorized as Q3, and those with a 3D-HLCs/2D-HLCs ratio > 2.0 were assigned to Q4. Proteins with a 3D-HLCs/2D-HLCs ratio > 1.5 were considered as upregulated proteins. **B**, Gene ontology (GO) terms annotated biological process, cellular component, and molecular function in differentially modified acetyl-proteome. **C**, Screening of upregulated target proteins with differential acetylation modifications based on 3D-HLCs/2D-HLCs ratio, with a ratio (fold change) > 1.5 defined as upregulated proteins. **D**, Protein-protein interaction network analysis of target proteins with differential acetylation modifications using the STRING (v.11.0) protein-protein interaction database. **E**, Western blot showed the protein expression of ALB, AFP, and acetylation levels of H3K56 in 2D-HLCs and 3D-HLCs. **F**, Immunofluorescence assay showed acetylation levels of H3K56 in 2D-HLCs and 3D-HLCs. **G**, The sonicated shearing DNA fragments in 2D and 3D-HLCs detected by using agarose gel electrophoresis. **H**, The PCR products of ChIP-qPCR were subjected to agarose gel electrophoresis. **I**, Fold enrichment of H3K56ac at the ALB promoter in 2D-HLCs and 3D-HLCs. *P < 0.05. Data are presented as mean ± SD.

The modification of histone acetylation is known to regulate gene transcription activation at the epigenetic level.^26,49^ Based on our previous findings that the mRNA level of ALB in 3D-induced HLCs was significantly higher than that in 2D-induced HLCs, we conducted a proteomic analysis of acetylation modification in 2D and 3D-HLCs. The results indicated that the upregulated acetylation proteins, defined by cluster analysis based on the fold change of 3D-HLCs/2D-HLCs expression greater than 1.5, were mainly involved in cellular processes, metabolism, and biological regulation. Gene ontology analyses of these upregulated acetylation proteins also showed that they enhanced chromatin binding and transferase activity, transferring acyl groups (Fig. 4A, 4B). Analysis of the histone H3 acetylation sites differentially expressed showed that H3K56ac was upregulated in 3D-HLCs, and the acetyltransferase p300 was also upregulated (Fig. 4C). These differentially acetylation modified proteins were further analyzed using the STRING (v.11.0) protein-protein interaction database, and histone H3 protein was found to have a direct interaction with p300 protein (Fig. 4D).

Western blot and immunofluorescence assay confirmed that the acetylation level of H3K56 in 3D-HLCs was significantly higher than that in 2D-HLCs, and the protein expression of ALB was also significantly higher in 3D-HLCs compared to 2D-HLCs (Fig. 4E, 4F). This suggests that H3K56ac may be a key feature of 2D and 3D-HLCs. To observe the enrichment of H3K56ac on the ALB gene promoter in HLCs induced by 2D and 3D hADMSCs, we performed a ChIP-qPCR method to detect the efficiency of H3K56ac enrichment on the promoter of the ALB gene in 2D and 3D-HLCs. The results showed that the enrichment of H3K56ac on the ALB gene promoter was significantly higher in 3D-HLCs compared to 2D-HLCs (Fig. 4G-4I).

Overall, these results suggest that, compared to 2D-HLCs, 3D-HLCs exhibit upregulated mRNA expression of ALB, which is attributed to the increased H3K56 acetylation level, leading to the activation of ALB transcription.

### A-485 Suppressed Hepatogenic Differentiation of hADMSCs by Inhibiting H3K56 Acetylation

**Figure 5.**
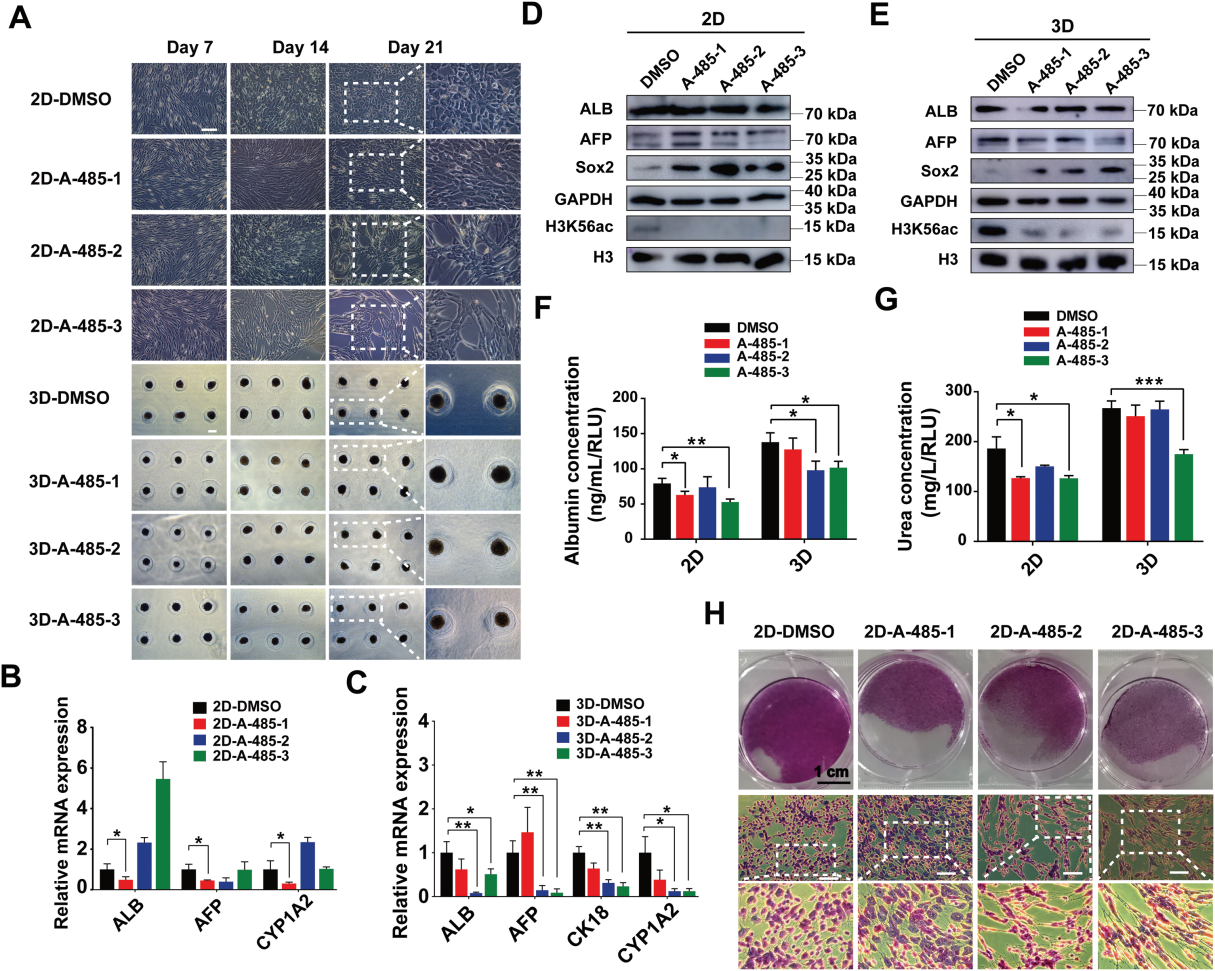
A-485 suppressed hepatogenic differentiation of hADMSCs in 2D and 3D cultures. p300 inhibitor A-485 (3 μM) was added in HLCs maturation stage (days 7-21) during 2-step process. A-485-1 group was A-485 (3 μM) was added on days 7-14 during HLCs maturation stage, A-485-2 group was A-485 (3 μM) was added on days 14-21 during HLCs maturation stage, A-485-3 group was A-485 (3 μM) was added on days 7-21 during HLCs maturation stage. **A**, Cell morphological changes of DMSO control and addition of A-485 at different time points during hepatic induction of hADMSCs. Scale bar = 100 μm. **B**, The mRNA expression of liver-related genes in DMSO and A-485 groups after 2D hepatic induction. **C**, The mRNA expression of liver-related genes in DMSO and A-485 groups after 3D hepatic induction. *P < .05, **P < .01 vs DMSO group. **D**, The protein expression of ALB, AFP, Sox2, and H3K56ac in DMSO and A-485 groups after 2D hepatic induction. **E**, The protein expression of ALB, AFP, Sox2, and H3K56ac in DMSO and A-485 groups after 3D hepatic induction. **F**, The albumin secretion of DMSO and A-485 groups after 2D and 3D hepatic induction. *P < 0.05, **P < .01 vs DMSO group. **G**, The urea production of DMSO and A-485 groups after 2D and 3D hepatic induction. *P < .05, ***P < .001 vs DMSO group. **H**, The PAS staining of DMSO and A-485 groups after 2D hepatic induction. Scale bar = 100 μm. Data are presented as mean ± SD.

Proteomic analysis revealed differential expression of p300, a histone acetyltransferase that regulates histone acetylation.^42,50^ H3K56 and p300 were found to interact in a protein-protein interaction network. To confirm whether H3K56 acetylation affects the hepatogenic differentiation of hADMSCs regulated by p300, the p300 inhibitor A-485 was used at different time points during the 2D and 3D induction maturation stage to investigate the optimal inhibitory effect of A-485. As shown in Fig. 5A, in the 3D induction, there were no significant changes in cell morphology, while significant changes were observed in the 2D-induced cells. When treated with A-485 from days 7 to 21, the A-485-2 and A-485-3 showed significant changes in cell morphology with changing from round irregular polygons to spindle-like shapes resembling mesenchymal stem cells. RT-qPCR and western blot analysis were performed to investigate the effects of A-485 at different time points on mRNA and protein expression of liver-related genes. In the 2D induction, the A-485-1 showed significant downregulation of mRNA expression of ALB, AFP, and CYP1A2, while the A-485-3 in the 3D induction showed significant downregulation of mRNA expression of ALB, AFP, CK18, and CYP1A2. Both 2D and 3D inductions showed that only the A-485-3 showed simultaneous downregulation of the protein expression of ALB and the acetylation of H3K56. Interestingly, we found that the A-485 could upregulate the protein expression level of stemness gene Sox2 (Fig. 5B-5E). In addition to observing cell phenotypes, the functions of albumin secretion, urea production, and glycogen synthesis were also measured in 2D and 3D HLCs. The results revealed that compared with the DMSO group, the A-485-3 significantly reduced the secretion of albumin and urea and glycogen synthesis in both 2D-HLCs and 3D-HLCs (Fig. 5F-5H). In summary, these results showed that the A-485-3 group significantly suppressed hepatogenic differentiation of hADMSCs by inhibiting H3K56 acetylation. Therefore, we selected the A-485-3 group as the optimal inhibition group for hepatogenic differentiation of hADMSCs in 2D and 3D for further research.

### p300 Affected Hepatogenic Differentiation of hADMSCs by Regulating H3K56 Acetylation

**Figure 6.**
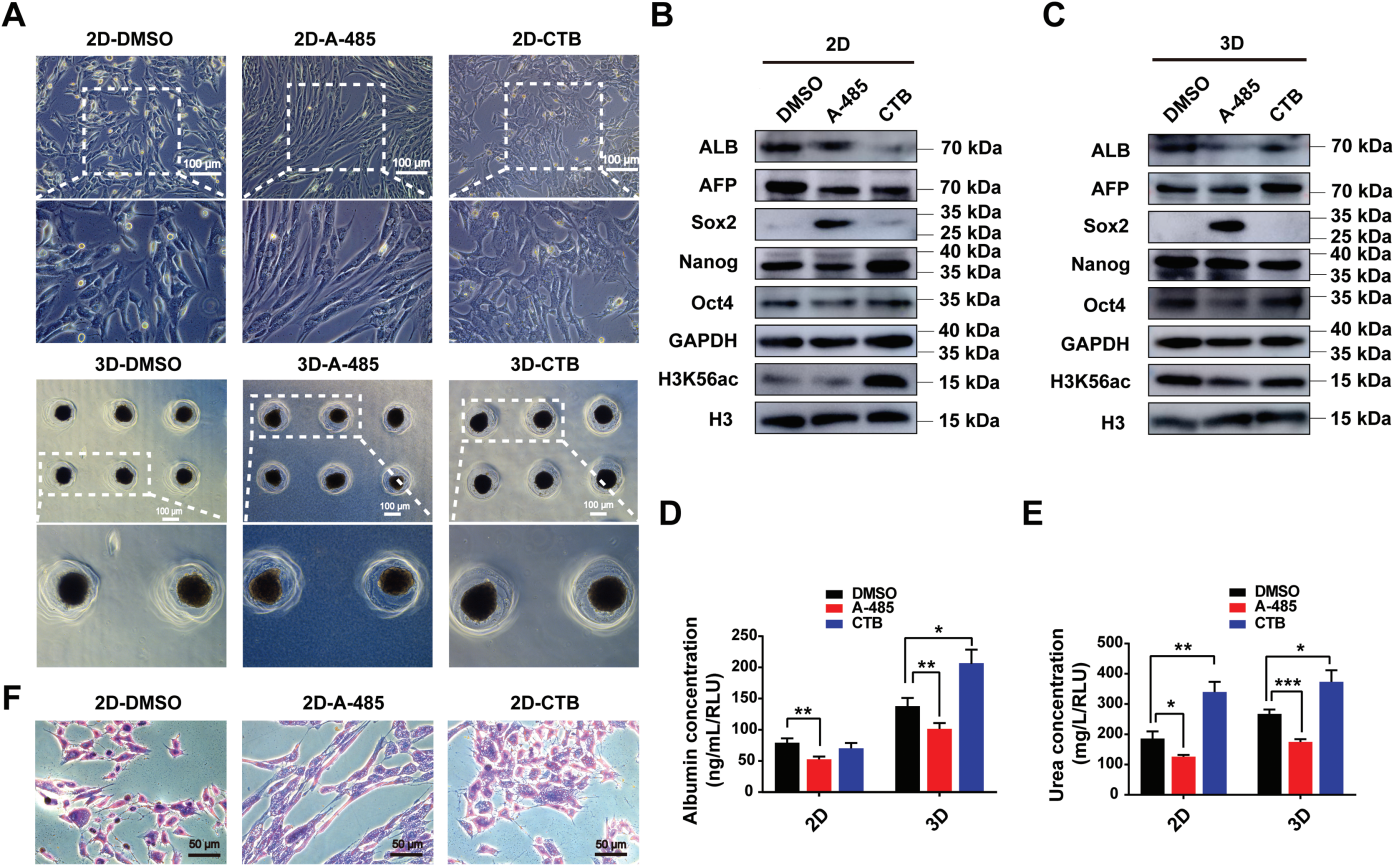
p300 inhibitor A-485 and activator CTB affected the phenotypic and functional changes of HLCs differentiated from hADMSCs in 2D and 3D cultures. A-485 (3 μM) was added on days 7-21 during HLCs maturation stage, which was A-485-3 group mentioned above. CTB (10 μM) was added in HLCs maturation stage (days 7-21) **A**, The morphological changes of cells with the addition of A-485 and CTB during hepatic induction of hADMSCs. **B**, The protein expression of related genes in DMSO, A-485, and CTB groups of 2D induction. **C**, The protein expression of related genes in DMSO, A-485, and CTB groups of 3D induction. **D**, The albumin secretion and **E**, urea production of DMSO, A-485 and CTB groups in 2D and 3D induction. *P < .05, **P < .01, ***P < .001 vs DMSO group. **F**, The PAS staining of DMSO, A-485 and CTB groups in 2D induction. Data are presented as mean ± SD.

To further confirm the effect of p300 on hepatogenic differentiation of hADMSCs through the regulation of H3K56 acetylation, we also added the p300 activator CTB to observe its effect on hepatogenic differentiation. While there were no significant changes in cell morphology in all groups for 3D induction, for 2D induction, A-485 significantly inhibited the transformation of cells into polygonal liver-like cells, while CTB promoted the transformation of cells into polygonal cells (Fig. 6A). In addition to observing the effects of p300 inhibition and activation on hADMSCs 2D and 3D differentiation based on cell morphology, we also measured the expression of relevant proteins in each group. The results, as shown in Fig. 6B, 6C, indicated that compared to DMSO, both 2D and 3D A-485 significantly decreased the acetylation level of H3K56 and the protein expression of ALB. In 2D induction, CTB upregulated the acetylation of H3K56 but downregulated the protein expression of ALB and AFP, while there was no significant difference in H3K56 acetylation in the 3D-CTB group. In addition to detecting the expression of liver-related proteins, we also examined the protein expression of pluripotency genes Sox2, Nanog, and Oct4. The results showed that compared to the DMSO control group, both 2D and 3D A-485 groups significantly increased the protein expression of Sox2. We also tested the secretion of albumin and urea and glycogen synthesis. Albumin ELISA and urea QuantiChrom analysis showed that compared to DMSO, A-485 significantly decreased the levels of albumin and urea in both 2D and 3D induction, while CTB significantly increased the level of urea in 2D and 3D induction and increased the level of albumin in 3D induction (Fig. 6D, 6E). PAS staining showed that A-485 significantly decreased glycogen synthesis, while CTB increased glycogen synthesis (Fig. 6F). Overall, these phenotypic and functional data revealed that p300 affected hADMSCs 2D and 3D hepatogenic differentiation by regulating H3K56 acetylation.

## Discussion

The 3D spheroid culture of mesenchymal stem cells (MSCs) has been shown to enhance their pluripotency and differentiation abilities compared to 2D monolayer culture.^24,51-53^ However, the epigenetic mechanisms underlying the impact of different culture dimensions on hepatogenic differentiation of human adipose-derived MSCs (hADMSCs) have not been clearly elucidated yet. In this study, we report a crucial role of acetylation modification of H3K56 in regulating hepatogenic differentiation of hADMSCs in 2D and 3D cultures. We propose a mechanism in which p300-mediated H3K56 acetylation promotes the differentiation of hADMSCs into hepatocyte-like cells (HLCs) in 3D spheroid differentiation.

Recently, ample researches have indicated that MSCs exhibit multilineage differentiation potential,^54-57^ such as osteogenic, adipogenic, and chondrogenic differentiation. Our study also showed that hADMSCs have the potential to differentiate into osteocytes and adipocytes. The pluripotency transcription factors Sox2, Oct4, and Nanog are important transcription regulatory networks in stem cells, playing a crucial role in multi-lineage differentiation and self-renewal.^58,59^ It was reported that 3D spheroids can partially simulate the oxygen and nutrient concentration gradient in the liver lobule from the portal vein area to the central vein, making it a good 3D culture model for hepatocytes.^60^ We found that 3D spheroid culture of hADMSCs increased the expression of pluripotency transcription factors Sox2, Oct4, and Nanog, compared to 2D culture. Moreover, 3D spheroids differentiation enhanced the phenotypes and functions of HLCs, further promoting the maturation of HLCs.

Epigenetic regulation plays a crucial role in the hepatogenic differentiation of stem cells. Recent studies have demonstrated that DNA methylation, as an epigenetic mechanism, can control self-renewal and lineage differentiation in stem cells, offering an alternative pathway to promote hepatic differentiation.^33,61^ Furthermore, it has been reported that long noncoding RNAs (lncRNAs) are involved in regulating the differentiation of human pluripotent stem cells into hepatic lineage progenitors.^62^ Another recent study highlighted the significance of histone crotonylation modification in promoting the hepatic differentiation of human embryonic stem cells.^63^ Additionally, histone acetylation, alongside the mentioned epigenetic mechanisms, also plays a crucial role in the pluripotency and differentiation of stem cells.^64^ Histone acetylation is a key epigenetic modification involved in gene transcription regulation and DNA repair, regulated by histone acetyltransferases (HATs) and histone deacetylases (HDACs).^29^ Recently, many studies suggest that HDAC inhibitors promote hepatic differentiation of MSCs, indicating that histone acetylation promotes hepatic differentiation of human MSCs.^33,65^ A study has shown that under 3D culture condition, increased cell-cell and cell-matrix interactions may promote pluripotent gene expression in MSCs through the relaxation of actin cytoskeleton, potentially leading to changes in histone H3 modification levels.^66^ Our data showed that 3D induction upregulated the acetylation of H3K56, enhanced the enrichment of H3K56ac on the promoter region of ALB gene, promoted ALB transcription, and upregulated the protein expression of ALB. Transcriptional coactivators p300 and CBP regulate gene expression and are involved in differentiation, as one type of HATs, p300/CBP regulates acetylation of H3K9, H3K14, H3K56, and so on,^67^ playing an important role in osteogenic and chondrogenic differentiation of MSCs.^68^ In this study, we showed that treatment with A-485, the p300 inhibitor, significantly inhibited cell morphology transition, decreased the acetylation level of H3K56 and the protein expression of ALB in both 2D and 3D induction. Furthermore, we observed that the mRNA expression of ALB and AFP in both 2D and 3D environments was influenced by the different A-485 treatment groups. This can be attributed to variations in cell-cell and cell-ECM interactions in these culture environments,^32^ as well as the impact of cellular architecture on mRNA expression.^69^ It is worth noting that the levels of mRNA and protein expression may not always correlate in 2D and 3D culture environments. This inconsistency can be attributed to various factors, including post-transcriptional regulation, protein degradation, and post-translational modifications.^70,71^A study reported that spheroid culture increased the pluripotency of human mesenchymal stem cells (hMSCs) and altered the epigenetic status of pluripotent genes. Specifically, the levels of histone H3 acetylation at K9 in the promoter regions of pluripotent genes Sox2, Oct4, and Nanog were found to be elevated.^31^ However, as the differentiation process progressed, the pluripotent genes were gradually silenced, while lineage-specific genes were activated.^72^ In this study, we also found that A-485 increased the protein expression level of Sox2, indicating the pluripotency and differentiation of stem cells are mutually inhibited and balanced during differentiation process, with activation of stemness gene expression and inhibition of differentiated cell-specific gene expression, which is consistent with previous study.^73^ Our study showed that A-485 also significantly decreased albumin, urea secretion, and glycogen synthesis. On the contrary, the p300 activator CTB promoted cell morphology transition, increased the acetylation level of H3K56 in 2D induction, but showed no significant change in 3D induction. CTB also significantly increased albumin, urea secretion, and glycogen synthesis. Thus, the findings of the current study suggested that p300 modulates hADMSCs hepatogenic differentiation in 2D and 3D cultures through regulating the level of H3K56 acetylation, and 3D differentiation enhances the efficiency of hepatogenic differentiation of hADMSCs by upregulating the acetylation of H3K56 and activating ALB transcription.

## Conclusion

In conclusion, our study provides a more precise epigenetic mechanism for MSCs hepatogenic differentiation and suggests that p300 affects hADMSCs 2D and 3D hepatogenic differentiation by regulating H3K56 acetylation. 3D spheroids culture facilitates differentiation of hADMSCs into HLCs through p300-mediated H3K56 acetylation. Further studies are required to elucidate the signaling pathways involved in p300-mediated H3K56 acetylation promoting hepatogenic differentiation of hADMSCs, providing a better understanding of MSCs hepatogenic differentiation and improving the hepatogenic differentiation strategy for potential clinical applications in liver regeneration and disease modeling.

## Data Availability

The data underlying this article will be shared on reasonable request to the corresponding author.
